# Innovations in peer review in scholarly publishing: a meta-summary

**DOI:** 10.12688/wellcomeopenres.17715.1

**Published:** 2022-03-09

**Authors:** Helen Buckley Woods, Johanna Brumberg, Wolfgang Kaltenbrunner, Stephen Pinfield, Ludo Waltman

**Affiliations:** 1 Research on Research Institute and Information School, The University of Sheffield, Sheffield, South Yorkshire, S10 2TN, UK; 2 Volkswagen Stiftung, Hannover, Germany; 3 Research on Research Institute and Centre for Science and Technology Studies, Leiden University, Leiden, The Netherlands

**Keywords:** peer review, scholarly publishing, innovation, meta-summary, review of reviews

## Abstract

**Background:** There are currently numerous innovations in peer review and quality assurance in scholarly publishing. The Research on Research Institute conducted a programme of co-produced projects investigating these innovations. This literature review was part of one such project ‘Experiments in peer review’ which created an inventory and framework of peer review innovations. The aim of this literature review was to aid the development of the inventory by identifying innovations in peer review reported in the scholarly literature and by providing a summary of the different approaches.

**Methods:** This meta-summary is based on data identified from Web of Science and Scopus limited from 2010 to 2021. A total of 247 papers were screened, with 6 review articles chosen for the focus of the literature review. Items were selected that described approaches to innovating peer review or illustrated examples.

**Results:** The summary of innovations are drawn from 6 review articles. The innovations are divided into three high-level categories: approaches to peer review, reviewer focussed initiatives and technology to support peer review with sub-categories of results presented in tabular form and summarised. A summary of all innovations found is also presented.

**Conclusions: **From a simple synthesis of the review authors’ conclusions, three key messages are presented: observations on current practice; authors’ views on the implications of innovations in peer review; and calls for action in peer review research and practice.

## Introduction

The Research on Research Institute (RoRI) conducted a number of co-produced projects with academic publishers and scholarly communication service providers. These projects investigated current experiments and innovations in quality assurance and peer review in scholarly publishing. This literature review is part of one such project entitled ‘Experiments in Peer Review’.

The ‘Experiments in Peer Review’ project aimed:

- to identify, analyse, and evaluate current innovations in peer review and other forms of quality control/assurance of research outputs
- to assess their potential impacts on scholarly communication in particular and the research environment in general.

The first phase of this project was to create an inventory and framework of experiments in peer review carried out by publishers and other scholarly communication organisations. The inventory is based on a widely distributed survey of scholarly publishers designed to retrieve information on current innovations at grass roots level ( Kaltenbrunner *et al.*, 2022). The purpose of this literature review is to provide context and aid the development of the inventory. To do so, we identify publications reporting innovations or experiments with peer review in scholarly publishing and we create a summary of the different types of initiative as identified in the literature.

### Definitions and framing

In this review the definition of peer review reflects the use of the term in the literature selected. That is an inclusive and broad interpretation of the phrase to include many aspects of evaluation and quality assessment. We have adopted a broad working definition of what constitutes a ‘peer’ to mean those with expertise or significant interest in a topic. Besides researchers this also includes other stakeholders or their agents within the system such as copyeditors and formatters, artificial intelligence (AI) software, members of the public, patients, advocates and lobbyists. By using the term ‘peer’ in this broad sense, peer review also encapsulates activities designed to ensure research integrity such as plagiarism checks and monitoring compliance with data management policies. Similarly, peer review also includes informal responses, questions and comments posted on social media, pre-print servers, e-journals or other places online in response to a given research output. These types of informal responses were found in a study examining disciplinary knowledge production ( Woods, 2018), where examples of researchers’ peer review practice were identified. The results of group interviews with researchers in applied fields identified that several other types of ‘peer review’ which did not involve scientific experts, but members of the public, those from other professions, advocacy and lobbying groups were also common occurrences. Over 20 years ago Barnett (2000) spoke about knowledge becoming a commodity tested by consumer reaction. Particularly in applied fields, this is coming to fruition, with this type of research having a greater number of consumers, invoking greater and different types of reaction and review.

The scope of this review is restricted to peer review in scholarly publishing, although similar observations about the benefits and limitations of peer review are found in publications concerned with other parts of the research system. One example is the onerous nature of peer review, which is a factor also associated with peer review in research funding ( Bendiscioli & Garfinkel, 2021), and research assessment ( Wilsdon, 2015), in addition to scholarly publishing ( Smith, 2006). It is also worth noting that this literature review is concerned with what people are doing, and what the innovations entail rather than why they are doing it. The motivations giving rise to innovation, for example to reduce bias or achieve greater efficiency are peripheral to the purpose of this study.

This literature review describes innovations. In a similar treatment to the interpretation of the term ‘peer review’ we adopt a broad interpretation of the term ‘innovation’. The definition proposed by Rogers (2003) is fitting for this review: ‘an idea, practice, or object that is perceived as new by an individual or other unit of adoption’ (p. 26). That is, the status of something as an innovation does not rest on the date of its inception, it could be in existence for 4 hours or 40 years, what makes it an innovation is if the practice is new to those who are implementing it. Within the academic publishing industry, what may be considered an innovation in one organisation, such as reviewers and authors being blind to each other’s identity, would no longer be considered an innovation in another. Broadly speaking, an innovation can be implemented in different ways within an organisation: first, by intervening in usual practice; second, by intervening on a smaller scale, in one area of work, to test something out before implementing it more widely; or third, in setting up a separate innovative project or initiative, outside existing processes. In this review all types of innovations are included, and the review is agnostic to breadth of implementation. All types of innovations are captured that were reported in the included studies. The next section will describe the methodology used in this literature review and how we will present the results. This is followed by the results themselves in which we classify different types of innovations. We conclude with some discursive reflections on the current situation.

## Methodology

### Overall approach

This review was undertaken to set the context and inform an empirical study ( Kaltenbrunner *et al.*, 2022). To complete this task, it was not necessary to identify every publication discussing peer review, rather to capture as many different forms of peer review discussed as possible. With this in mind we did not limit the search to a particular publication type and our search results included several review articles. On closer inspection of these articles, it was clear that they covered all the peer review innovations identified from screening and coding the results of the literature search. This is with the possible exception of modelling or scientometric studies examining aspects of the peer review system ( Ortega, 2017; Ragone *et al.*, 2013) or proposing a framework for best practice for academic publishing ( Waters *et al.*, 2020) or audit of publishing processes ( Crewe, 2020). However, these papers were slightly out of scope for the remit of the empirical work. Therefore, the decision was made to present the data through the organising structure of six recent literature reviews on the topic, loosely based on the methods for presenting an “umbrella review” ( Aromataris *et al.*, 2015). That is to say, the synthesised findings of each review are presented and combined to create a meta-summary of peer review types, rather than describing disaggregated findings from the primary studies included within each review. This was a pragmatic decision and was not intended to detract from primary studies such as Walker and Rocha da Silva (2015) which presents a thorough and useful overview of peer review types, which is cited by Tennant *et al.* (2017); Horbach & Halffman (2018); Tennant (2018); and Barroga (2020) in this meta-summary.

Of high importance to this review and RoRI’s work with scholarly publishers is the Reimagine Review registry set up by Accelerating Science and Publication in biology (ASAPbio). The projects included in this registry provide live examples of the types of peer review innovations summarised in this review, such as post-publication review and pre-print review. More details of the registry are provided in Box 1.

**Box 1. ASAPbio’s Reimagine Review**

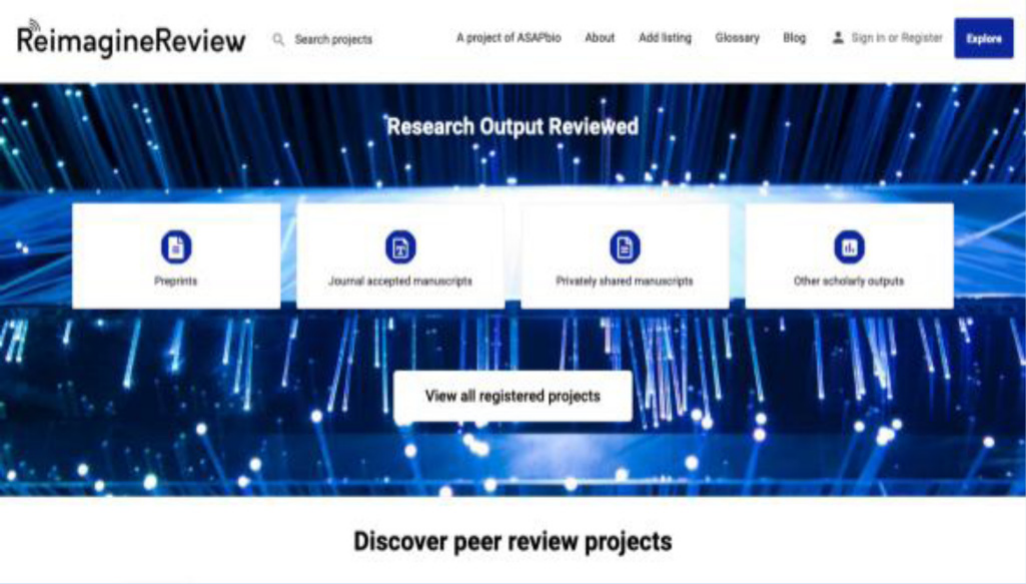

Reimagine Review is a registry of peer review experiments. As of December 24, 2021, it includes 59 registered projects.

The registry is presented as a searchable database. The user is able to filter the records in various ways including type of output, who initiates the review, whether the reviews are stand alone or linked to a specific publication, the level of transparency or ‘openness’, whether a decision is made at the end of the review (to publish or not), discipline, format of reviewing (such as comments or scores) and some characteristics of the process (for example, if professional editors are used, if comments are moderated). Alternatively, the top page enables authors to choose by output: pre-print, articles already accepted for publication by a journal, privately shared manuscripts and finally ‘other outputs’ such as protocols, data sets etc. This enables authors to choose the most appropriate service that fits with their needs. The types of innovation featured in the Reimagine Review inventory ( ASAPbio, 2021) such as post-publication review have been included in this review.

### Data collection

A literature search was conducted in Web of Science and Scopus to identify relevant papers using synonyms for ‘peer review’ and ‘innovation’. With duplicates removed this produced 247 papers. Papers were screened to include only studies that described a type of innovation in peer review, or gave an example of a specific innovation. Papers describing concerns with peer review or the history of peer review were not included. After screening, the resultant 37 relevant papers were used to identify citing articles. The citation searches produced 44 results which were also screened and reduced to 31 articles. The final number of initially included papers was 68. These were reviewed and six review articles were chosen for the focus of the literature review. Searches were conducted in January 2021, limited from 2010 and no study filters were applied. An example search strategy is presented below.

### Example search strategy

***Web of Science via Clarivate.*** TI=("peer reviewing" OR "peer reviewer" OR "peer review ") AND TI=(experiment* or pilot* or improvement* or innovation* or solution* or initiative* or intervention*)

Timespan: 2010-2021. Indexes: SCI-EXPANDED, SSCI, A&HCI, CPCI-S, CPCI-SSH, BKCI-S, BKCI-SSH, ESCI.

### Study selection / coding

Search results were initially downloaded to EndNote (X9.3.2) to facilitate de-duplication after which study selection was completed in the Rayyan software Ouzzani *et al.* (2016). This allowed easy viewing of decision making by the project team. Initial categories of innovations were developed to include overview articles, types of peer review, reviewer focussed initiatives, technological initiatives and specific uses of peer review (such as use of language or plagiarism). This exercise of developing topic categories aided the organisation of material in the review. As previously stated, several review articles were identified in this process and on closer inspection of these articles, it was clear that they covered all the peer review innovations identified from screening and coding the results of the literature search. The review therefore focussed on six literature reviews in a review of reviews format. Please note an earlier version of this article can be found on SocArXiv (doi: 10.31235/osf.io/qaksd).

## Results

### Presentation of results

**Figure 1.**
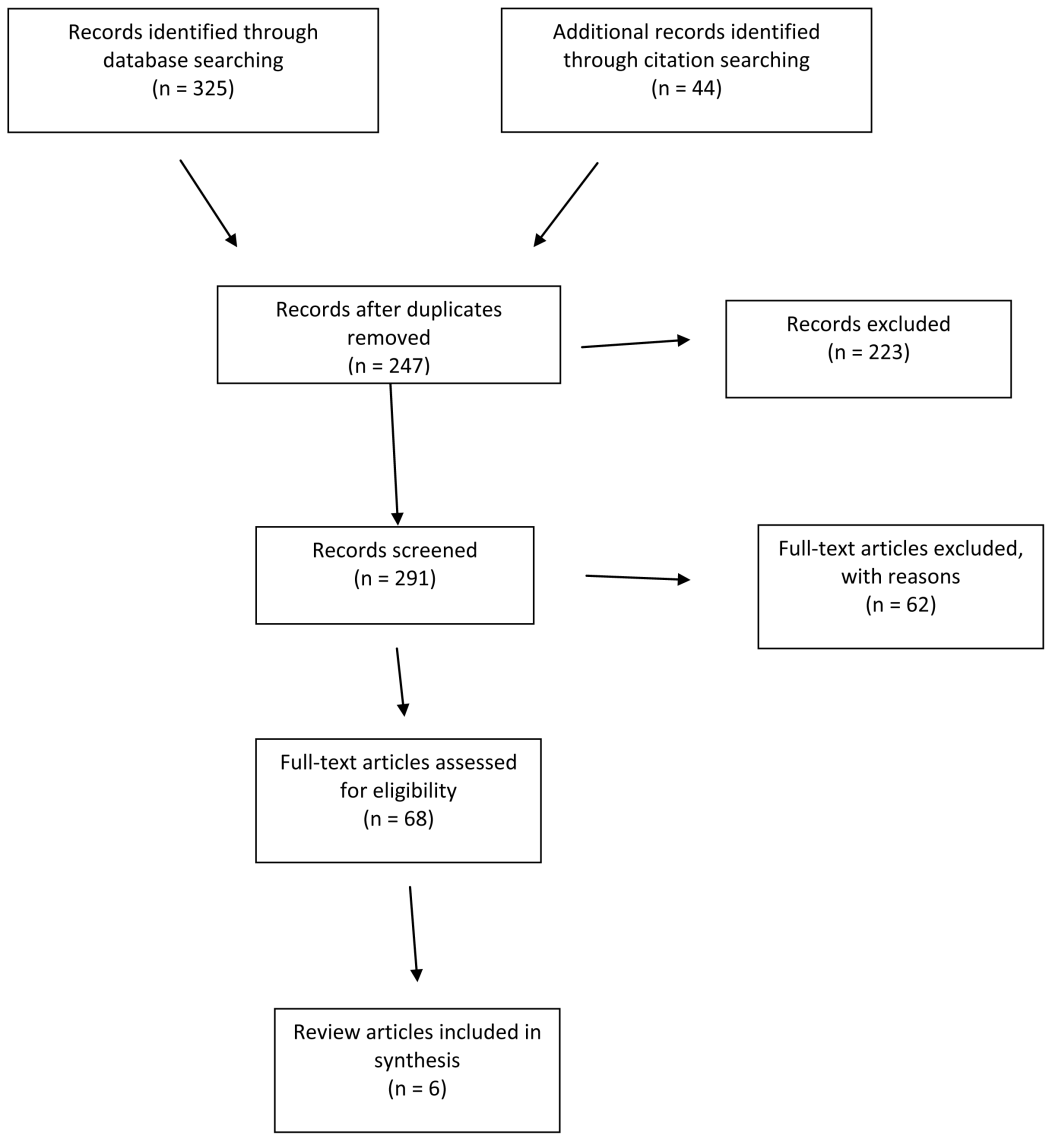
Flow chart based on PRISMA diagram (2009).

The results are presented using narrative and tabular formats, followed by a summary of the review and conclusions. The results section begins with a description of included studies, followed by a detailed description of innovations in peer review types using three high level categories: approaches to peer review, reviewer focussed initiatives, and technology to support peer review. This includes definitions of each type of innovation extracted from the included studies. A summary table of each innovation and where these have been reported is provided at the end of the review. See Figure 1 for a PRISMA diagram giving details of the search process.

### Details of included studies

The review includes six overview studies which range from a systematic review of randomised controlled trials to a succinct summary of review innovations. All the studies were valuable in capturing the different types of current innovation in peer review. Each study is described below, presented in chronological order. This is followed by a narrative summary of each type of innovation, followed by a summary table, giving definitions of each innovation and citations to the respective studies where they are found.

Bruce *et al.* (2016) is a systematic review and meta-analysis published in *BMC Medicine* which aims to evaluate the effectiveness of innovations to improve the quality of peer review for publications in biomedical science. It includes 22 randomised controlled trials.

The innovations that were evaluated were: reviewer training, addition of a statistical peer reviewer, open peer review (where reviewers’ identity is known), blinded peer review (where reviewers’ or authors’ identity is not known), and innovations to increase the speed of peer review. The unit of randomisation were either peer reviewers or manuscripts depending upon the innovation being assessed, for example comparing the effect of adding a statistical reviewer against the usual process of peer review in a given set of manuscripts. For details of study characteristics see Bruce *et al.* (2016). The review was based on a search of CENTRAL, MEDLINE (PubMed), Embase, Cochrane Database of Systematic Reviews, and WHO ICTRP databases. Several scales were used by the authors, such as final manuscript quality ( Goodman *et al.*, 1994) and quality of the peer review report ( Black *et al.*, 1998) to assess the different outcome measures: final manuscript quality, quality of the peer review report, rejection rate, time spent on peer review, and time spent on the peer review process. The authors found, based on these outcome measures, that compared with standard peer review, reviewer training was not successful in improving the quality of the peer review report and use of checklists by peer reviewers to check the quality of a manuscript did not improve the quality of the final article. However, the addition of a specialised statistical reviewer did improve the quality of the final article and open peer review was also successful in improving the quality of review reports. It did not affect the time reviewers spent on their report. Open peer review also decreased the number of papers rejected. Finally, blinded peer review did not affect the quality of review reports or rejection rates. The authors conclude that there is a lack of evidence on the effectiveness of various peer review procedures, especially given its central role in science.

Tennant *et al.* (2017) is a narrative review published in *F1000 Research*. Its process is notable as the review has 33 authors. They are experts in scholarly publishing, and it is these expertise which formed the basis of the review. The authors identified papers through searching databases such as Google Scholar, Scopus, and Library & Information Science Abstracts (LISA). Additionally, there was a lengthy peer review process which was published with the review including reviewers reports and authors responses, a key part of the F1000 model (and itself an innovation in peer review). The authors review the history of peer review, its myriad shortcomings and details the potential solutions that have been developed to date to address these challenges. The pros and cons of newer innovations in peer review such as portable, de-coupled and collaborative peer review are then discussed as well as an examination of different levels of anonymity. They then go on to focus on a number of social web platforms, such as Reddit and expand on the benefits and limitations of each platform considering three criteria: quality control and moderation, certification, and incentive structures. Alongside the review of new innovations, the authors are clear to signal that there are particular benefits of peer review and that it has deep and far-reaching cultural significance within research practices which should not be underestimated. A hybrid model is suggested combining aspects of different platforms. The authors stress that any such innovation cannot succeed without engagement from researchers, but this is in tension with the structure of researcher incentives in the research system. The review concludes with two main points, one to decouple peer review from journal publishing in order to return to what the authors suggest would be a community-led process. Secondly, there is very little evidence to support the uptake of different methods of peer review, so research to measure the effectiveness of these different approaches in achieving different goals of peer review is essential. Three key initiatives are referred to as leaders in this respect: the PEERE initiative, the *Research Integrity and Peer Review* journal, and the International Congress on Peer Review and Scientific Publication.

Burley (2017) is a summary article published in *Information Services and Use* which reviews new approaches to peer review in scholarly publishing. The author is affiliated to the BMC Group at the Springer Nature Publishing Company. The article does not refer to scholarly literature, but refers to new innovations and ways of working in practice. The article begins by stating the benefits of peer review by key stakeholders. The author then goes on to summarise newer practices such as the increase in double-blind peer review in scientific disciplines. She then discusses open peer review, post-publication peer review and transparent peer review. Finally, initiatives aimed at increased efficiency such as cascading peer review and sectional or partial peer review are discussed. The article concludes by highlighting the increased focus of rewarding and training reviewers by scholarly publishers and learned societies and the overall improvement in reviewer recognition and efficiency, as well as the increase in experiments in transparent peer review.

Horbach & Halffman (2018) is a narrative review published in *Research Integrity and Peer Review*. It aims to describe current forms of peer review and their implementation and also consider the role and expectations of peer review. The authors present an historical account of peer review and describe methods of peer review to date, including recent technological advances. Four dimensions are identified in peer review innovations: ‘the selection conditions [such as the timing of the review], the identity and access among actors involved, the level of specialisation in the review process, and the extent to which technological tools have been introduced’ (p. 9). The authors then present a typology of peer review characteristics ordered by these four dimensions. This is followed by a discussion on the role of the academic publishing system and expectations of peer review. The authors underline the large diversity of review processes currently used. They also suggest four key expectations for peer review: ‘assuring quality and accuracy of research, establishing a hierarchy of published work, providing fair and equal opportunities to all actors and assuring a fraud-free research record’ (p. 12). The article concludes by highlighting the lack of empirical evidence to test the efficacy of peer review methods and the tensions that exist between what peer review can deliver and what is expected of it, for example its ability to identify fraudulent research or methodological errors. The authors suggest there is a new, additional perspective, in how research knowledge is perceived, fuelled by statistical reviews, post-publication reviews and other innovations, from a library of knowledge, to a set of scientific facts. They suggest that this perception of research as 100% accurate knowledge fuels retractions and rewriting of documents to create seemingly perfect accounts.

Tennant (2018) is a state-of-the-art review published in *FEMS Microbiology Letters*. Explicit methods are not stated. There are 87 references listed in the document. Commensurate with the aims of a state-of-the-art review ( Grant & Booth, 2009), this article centres on the current status of peer review and its role in scholarly publishing in a digital age. The author states a conceptual difference between peer review as an idea, or ‘a singular ideologue’ (p. 2) and a practice. Open peer review is cited as a return to the original purpose of peer review to be collegial, constructive, to improve arguments and gaps in logic. The author states that peer review now has an additional gatekeeping function, and it is also used by commercial organisations as a selling point. He goes on to summarise the benefits and drawbacks of new models of peer review considering what the job of peer review is and how these functions can be achieved in the future, making better use of the technology we now have. Success would be an open participative model of peer review that is a genuine alternative rather than an add-on to the status quo. However, the author also states that it is difficult to separate the value and prestige that comes from publishing in journals, and this drives particular behaviours and limits uptake of new models. He refers to the ‘penguin effect’ ( Choi, 1997) where the level of perceived risk is greater than the motivation to change. This effect is compounded by the fact that moving away from current practices is often not in sync with the behaviours required for researchers’ job security and career progression. The author concludes by advocating a new framework based on current technological and communication norms by revisiting the core purposes of peer review, links to incentives for researchers and a clear consideration of how all stakeholders fit into any new system.

Barroga (2020) is a narrative review published in the *Journal of Korean Medical Science* giving an overview of innovations in peer review. The review is based on a search of MEDLINE, Embase and Scopus databases and uses the peer review innovations as stated in Tennant *et al.* (2017) to organise the material. The author begins by dividing peer review into two types: ‘open peer review’ and ‘traditional peer review’. He compares the features of these two review types, using various criteria such as openness, bias, time and so on. He then takes eleven different innovations stated in Tennant *et al.* (2017) and compares them against the same features. After comparing a number of web platforms/models of peer review (also from Tennant *et al.*, 2017) he briefly discusses delays to peer review and the issue of anonymity as manifested in ‘blinding’ of reviewers. Finally, the discussion turns to reviewer incentives and training. The author concludes that the increase of innovations has been rapid and there is a lack of evidence as to how effective newer methods of review are in identifying research malpractice. He also suggests that review quality may be compromised where financial incentives are given. He advocates for an honest appraisal of stakeholder’s contribution to the process with reviewer training, core competencies for reviewers and engagement by the research community on this issue being paramount.

See Table 1 for a summary of the general characteristics of the studies included.

**Table 1.** General characteristics of included studies.

Author / Year / Journal	Type of review	No of included studies (size of review)
Bruce *et al.* / 2016 / BMC Medicine	Systematic review and meta-analysis	22 randomised controlled trials
Tennant *et al.* / 2017 / F1000 Research	Narrative review	300
Burley / 2017 / Information Services and Use	Summary article	N/A
Horbach & Halffman / 2018 / Research Integrity and Peer Review	Narrative review	119
Tennant / 2018 / FEMS Microbiology Letters	State of the art review	87
Barroga / 2020 / Journal of Korean Medical Science	Narrative review	48

### Description of innovations in peer review

The innovations presented below are divided into three high-level categories: approaches to peer review, reviewer focussed initiatives and technology to support peer review. This section begins with narrative summaries of the different approaches to peer review identified, organised into the following subcategories: open / masked peer review; pre / post publication review; collaboration and decoupling; and focussed and specialised review. The descriptions will be followed by a summary table for each subcategory.

***Approaches to peer review.***

**Open/masked peer review**

All the studies in our review mention open peer review (OPR). The data reveals that it is not a clearly defined concept. However, understandings of OPR centre around the (i) identities of the reviewers, editors and authors being known to each other in various combinations or made public and (ii) reviewer reports and authors responses to comments being made public. Burley (2017) makes a distinction between the reviewer reports being signed, which she terms OPR, or not, which she terms transparent peer review. In his state-of-the-art review Tennant (2018) reflects a wider definition of OPR citing Ross-Hellauer (2017), who goes beyond transparency of identity to include other aspects of peer review, including open final version commenting, open pre-review manuscripts and open platforms. Finally, Tennant *et al.* (2017) refer to a survey of peer review stakeholders (OpenAIRE) which found 122 different definitions to be in use.

**Pre/post publication review**

The key feature of peer review innovations in this subcategory is the timing of the review. The authors’ definitions reveal a mixture of informal and formal peer review, open and confidential modes, expert and lay commentary. Barroga (2020) (after Tennant *et al.*, 2017) distinguishes between pre and post publication review and commenting. The key difference between review and commenting is who is responding to the publication. In pre and post publication review, this is field experts. In pre and post publication commenting, this amounts to comments or feedback by any interested party, irrespective of their academic or disciplinary credentials. Burley (2017) also describes post-publication review as taking place after publication, in an open manner. In an interesting use of the term ‘post-publication’ Horbach & Halffman (2018) describe post-publication peer review on pre-print servers. This clearly problematises the established use of the word ‘publish’ to mean publication in a journal or monograph. If something has been posted on a pre-print server, then it is published, albeit in a self-published mode, incurring only initial checks for eligibility to be posted on the particular pre-print service.

One other review innovation in this subcategory is the use of registered reports or similar approaches. This is where a research design is evaluated before the research has begun. It typically applies to quantitative empirical research that follows a fixed *a priori* design. Once a study is designed, the protocol is reviewed, before any data is collected. The value of this method is to reduce questionable research practices, where researchers deviate from their original intention and methodology and indulge in malpractice such as p-hacking and cherry-picking results to create more eye-catching conclusions. Registered reports are championed by the Center for Open Science amongst others.

**Collaboration and decoupling**

The approaches to peer review in this subcategory reflect a loosening of established roles and have been organised within a summary table ( Table 2) to illustrate this, with the more marked changes presented last. The types of peer review move from increased collaboration and interaction between stakeholders (collaborative review) to reassignment of roles in organisational innovations (decoupled post-publication review). Collaborative review ( Barroga, 2020) is the process where reviewers, editors and other contributors pool their comments to offer one set of consolidated recommendations for authors to address. Horbach & Halffman (2018) present a similar process which they name ‘discussion during review’. In a step further, this same process takes place online so other people can follow the process and add their own comments. Barroga (2020) and Tennant (2018) suggest the additional participants are limited to ‘other interested scientists’ but it is unclear how this can be enforced given the public platform.

**Table 2.** Approaches to peer review.

1. Open/masked peer review		
Label	Author	Description from paper
Open peer review	Barroga (2020)	‘Open peer review discloses the names of the editors and reviewers handling the paper to the authors.’
Open peer review	Bruce *et al.* (2016)	‘“Open” peer review process, whereby peer reviewers are informed that their name would be revealed to the authors, other peer reviewers, and/or the public.’
Open peer review	Burley (2017)	‘Open peer review with signed reports that are available with the published article, where a response by the author may be included.’
Transparent peer review	Burley (2017)	‘Transparent peer review by which the unsigned review reports are made available alongside the published article, and a response by the author may be included.’
Open review	Horbach & Halffman (2018)	‘We use the term ‘open review’ merely to indicate that the identity of the authors and reviewers are mutually known to each other.’
Open peer review	Tennant (2018)	‘It has been diagnosed to refer to seven key aspects of peer review: open identities, open reports, open participation, open interaction, open pre-review manuscripts, open final-version commenting and open platforms (or ‘decoupled review’) ( Ross-Hellauer, 2017)
Open peer review	Tennant *et al.* (2017)	‘No agreed definition.’ Cites OpenAIRE survey which found 122 different definitions in use ( Ross-Hellauer, 2017)
Blinded/masked peer review	Bruce *et al.* (2016)	‘... reviewers are blinded to author names and affiliation. Author names and/or potentially identifying credentials are removed from manuscripts sent for peer review so as to remove or minimize peer reviewer biases that arise from knowledge of and assumptions about author identities.’
2. Pre/post publication review		
Label	Author	Description from paper
Pre-peer review commenting	Barroga (2020); Tennant *et al.* (2017)	‘Pre-peer review commenting involves the informal commenting or discussion on a publicly available pre-publication manuscript draft.’
Pre-publication peer review	Barroga (2020); Tennant *et al.* (2017)	‘Pre-publication peer review consists of a formal and editorially invited evaluation of research by selected experts in the relevant field.’
Post-publication peer review	Barroga (2020); Tennant *et al.* (2017)	‘Post-publication peer review comprises a formal and optionally invited evaluation of research by selected experts in the relevant field after publication.’
Post-publication commenting	Barroga (2020); Tennant *et al.* (2017)	‘Post-publication commenting consists of an informal discussion of published research independent of any formal peer review.’
Post publication peer review	Horbach & Halffman (2018)	‘In these archives [pre-print servers], manuscripts usually go through a minor evaluation to check whether they meet minimal standards of academic writing. Subsequently, the actual review is done by community members who comment on the manuscript... Authors can then improve the manuscript and upload new versions to the archive.’
Post publication peer review	Burley (2017)	‘... takes place only after publication and is usually fully open.’
Registered reports	Horbach & Halffman (2018)	‘In this form of peer review, which is still restricted mainly to medical fields and psychology, manuscripts are usually reviewed in two stages. The initial and most important review stage takes place after the study has been designed, but prior to data collection.’
3. Collaboration and decoupling		
Label	Author	Description from paper
Collaborative review	Barroga (2020); Tennant *et al.* (2017)	‘Collaborative review involves manuscript assessment wherein referees, editors, and external readers provide interactive comments leading to a consensus decision and a single set of revisions.’
Interactive peer review	Barroga (2020); Tennant *et al.* (2017)	‘... the reviewers interact online with the authors and other interested scientists for a more open and collaborative review.’
Discussion during review	Horbach & Halffman 2018	‘Some journals … have attempted to improve editorial decision making by introducing interactive stages in the review process, during which reviewers and editors can share or discuss their reports and opinions on a manuscript before communicating a final decision to the author’
Cascading peer review	Barroga (2020); Tennant *et al.* (2017)	[Using this method] … ’rejections are avoided by redirecting peer-reviewed but rejected papers to a more suitable publication venue. The consortia enable papers with the referee reports to move easily between publishers, reducing time and expense of repeated evaluation. Some pass on the peer reviews with the rejected papers. Occasionally, reviews from other journals accompanying manuscripts rejected are used for other journals.’
Cascading peer review	Horbach & Halffman (2018)	‘[This model…is now widely used, especially by larger publishing houses. The system aims to avoid final rejection of a manuscript after peer review by redirecting critically reviewed manuscripts to potentially more suitable journals.’
Cascading peer review	Burley (2017)	‘[This method]…is based on the principle of reviewing a submitted manuscript only once (if possible) and offering to the author(s) a suitable publication venue in a tiered structure.
Peer review as a separate service	Burley (2017)	‘More experiments are underway [such as] making peer review independent of the journal and providing it as a service’
Recommendation services	Barroga (2020); Tennant *et al.* (2017)	‘Recommendation services review involves post-publication evaluation and recommendation of significant articles, often through a peer-nominated consortium.’
Portable review	Barroga (2020); Tennant *et al.* (2017)	‘Portable review means the authors pay a company...for a standard single-blind review that they can submit with the paper to collaborating journals.’
Independent peer review	Barroga (2020); Tennant *et al.* (2017)	‘In independent peer review … a number of companies provide pre-submission peer review for a fee...Thus, reports from commercial reviewer platforms are used to assist in peer review. This involvement of commercial refereeing bodies allows the dissociation of review from the journal publishing the article, thereby facilitating a faster review (e.g., Rubriq, Peerage of Science, Axios Review) or the detection of integrity issues (e.g., Research Square). Some companies use an online “scorecard” to determine strengths and weaknesses of a paper. For Peerage of Science, the fee is paid by the journal which publishes the offering.’
Decoupled post- publication review	Barroga (2020); Tennant *et al.* (2017)	‘Decoupled post- publication review consists of adding notes directly to the highlighted sections of the work. These added notes can be kept private or made public.’
Review by third parties	Horbach & Halffman (2018)	‘In addition to the systems providing pre-publication review, other independent platforms have emerged... in which any reader can comment on any published manuscript. These systems constitute examples of post-publication review independent of journals and publishers. These new trends have increasingly widened the definition of a peer, so that the term now refers … to anyone who feels capable of understanding and evaluating a given piece of research.’
4. Focussed and specialised review		
Label	Author	Description from paper
Soundness only review	Horbach & Halffman (2018)	‘A major development in [peer review models] … came with the launch of the open access journal PLoS ONE, by the Public Library of Science (PLoS), in 2006. In this journal’s review process and business model, reviewers are asked to base their recommendation for acceptance or rejection purely on the soundness and validity of the research, comprising the methodology, soundness of results and reporting.’
Results free review	Burley (2017)	‘Results-free review means that in a first step the paper is evaluated only for its rationale and method, not the results. If the former is deemed suitable for publication, then this is offered in principle. In a second step, the results are reviewed too. Publication may only be rejected if the results deviate unjustifiably from the stated aims and methods.’
Specialised review	Horbach & Halffman (2018)	‘Over the past decades, new actors have joined the review process, thereby compelling peer review itself to become more specialised. This applies to its content, for example introducing specialised statistical reviewers, as well as to the process ...’
Specialised review	Horbach & Halffman (2018)	‘... plagiarism detection software tool to assist in peer review, the CrossCheck system being the most common ...’ ‘Automatic analysis that checks for the correct use of statistics in manuscripts using AI.’
Specialised review	Horbach & Halffman (2018)	‘The assistance of software in detecting image manipulation, which is considered an increasing form of fraud in various research areas... it has already become possible to check for bad reporting, … data fabrication and image manipulation … usually done by … the editorial team or journal’s staff.’
Specialised review	Bruce *et al.* (2016)	‘Addition of peer reviewers for specific tasks or with specific expertise such as adding a statistical peer reviewer, whose main task is to detect the misuse of methods or misreporting of statistical analyses.’

Moving away from increased interaction as a focus of innovation and the slight modification of traditional procedures, the next type of innovation in this category is cascading or transferring peer review. This innovation was found three times in this review: Barroga (2020) (after Tennant *et al.*, 2017), Horbach & Halffman (2018), and Burley (2017). It is the process whereby an article that has already been peer reviewed and rejected by one journal is given the opportunity to be considered by another journal within the same publishing company. Barroga (2020) (after Tennant *et al.*, 2017) also suggests the process on a larger scale where publishers band together in consortia, enabling papers to move between journals owned by different publishers.

The final set of innovations in this subcategory reflect the deregulation of academic publishing as several new businesses emerge onto the market to provide peer-review services. This is reported by Burley (2017). Four variations on this theme emerge from Barroga (2020) (after Tennant *et al.*, 2017): recommendation services, portable peer review, independent peer review, and de-coupled post-publication review. A key aspect of all of these is that they are journal agnostic, that is, the process of peer review is not directly linked to a particular journal’s decision-making process in relation to the article. Recommendation is about promotion of particular articles that have been reviewed post-publication through a respected consortium of researchers such as F1000Prime, now Faculty Opinions ( Thorburn, 2020). As defined by Barroga (2020) (after Tennant *et al.*, (2017) portable peer review involves paying for an article to be reviewed and receiving the reports to submit to a publisher alongside the article. Independent peer review is again a commercial review company providing a service for an author, with the difference being that some publishers foot the bill for the review when a paper is subsequently published in their journal. De-coupled post-publication review is described by Barroga (2020) (after Tennant *et al.*, 2017) as articles being annotated online and notes added in the margins in either a private or a public mode. This fits with the broader sense of the term decoupling used in scholarly communication to mean the overall relaxation between peer review and dissemination ( Priem & Hemminger, 2012). Finally, Horbach & Halfmann (2018) highlight the open nature of some peer review services in their category ‘review by third parties’. This open nature enables anyone who feels they can comment on a piece of research the opportunity to do so, which they reflect ‘increasingly widen[s] the definition of a peer’.

**Focussed and specialised review**

This category captures types of peer review that focus on one aspect or section of a publication. Soundness only peer review ( Horbach & Halffman, 2018) refers to a method of reviewing in which only the rigour of the research (as opposed to its novelty or significance) is considered in making a decision on acceptance. It is akin to the critical appraisal method often adopted in reviewing health research for example in using a Critical Appraisal Skills Programme checklist ( CASP, 2018). The aim is to allow all results to be published which meet a particular quality threshold, not only the most interesting or novel results. ‘Results free peer review’ ( Burley, 2017) refers to a method of screening papers in a two-stage review process. This involves evaluating the rationale for the study and the methods. In the case of a positive evaluation, the paper is approved for publication in principle subject to a further full review that also includes the results. Horbach & Halffman (2018) and Bruce *et al.* (2016) both report instances of specialised review where a paper is reviewed with a focus on one aspect. This includes plagiarism detection, use of statistics and use of images. This work is done by various actors including researchers and editorial or journal staff, or by utilising specialist tools such as CrossCheck, a publisher initiative using the iThenticate text comparison software ( Feinstein, 2008). AI tools may also be used for this kind of work. Table 2 summarises the different types of peer review found in the literature.

***Reviewer focussed initiatives***

**Reviewer incentives**

This subcategory describes various incentives that are offered to induce researchers to act as peer reviewers. The incentives are manifest in direct and indirect rewards for peer review.

Direct rewards have been designed to reward peer review on top of the traditional indirect rewards. Direct rewards come in a range of forms, such as linking peer review to ORCID records, which Barroga (2020) (after Tennant *et al.*, 2017) terms ‘crediting’, or making peer review activity visible in Publons, a platform ‘dedicated to publicly recognising reviewers’ ( Burley, 2017). Financial reward (which can be private, or publicly acknowledged) is referred to by Barroga (2020) (after Tennant *et al.*, 2017), in the form of free access to articles, waivers of article processing charges, and fees for providing pre-publication review. Barroga (2020) states some difficulties with introducing payment for reviews, for example, commodifying peer review being in tension with academic culture. Tennant (2018) touches on the implicit / explicit and private / public nature of reward for peer review, commenting on the limitations of the scope of public reward due to the private nature of most peer review.

Indirect rewards are about being a good academic citizen, taking part in peer review as a usual part of academic work. These rewards are well established. As examples of indirect rewards of a private nature, Barroga (2020) mentions being up to date with one’s field and having the opportunity to influence the direction of the field. Other indirect rewards are of a public nature, such as being invited to be on an editorial board. Regardless of their private or public nature, indirect rewards do not bring an immediate benefit but instead help to promote one’s reputation, gain experience and contribute to the wider research system.

**Reviewer support**

Innovations to support reviewers include standards, training and tools for reviewers. Barroga (2020) cites informal training that researchers do for themselves such as reading instructions for authors, or asking colleagues for support. Also, training that is not designed specifically for reviewers but helps in a lateral way in undertaking the role, such as keeping up to date about advances in open access. In addition, he cites formal training courses set up by the Publons Academy. Bruce *et al.* (2016) also refers to reviewer training and mentoring programmes for peer reviewers to help them evaluate manuscripts appropriately. Core competencies for peer reviewers based on their responsibilities to readers, authors and editors are another form of reviewer support. These are based on the recommendations of particular associations such as the Council of Science Editors (CSE) and the Committee on Publication Ethics (COPE) ( Barroga, 2020). Finally, Bruce *et al.* (2016) reports on the use of checklists to aid peer review, such as reporting guidelines for different study types. Table 3 summarises the different types of reviewer focussed initiatives found in the literature.

**Table 3.** Reviewer focussed initiatives.

1. Reviewer incentives		
Label	Author	Description from paper
Non-financial	Barroga (2020)	’Nonfinancial incentives may come in the forms of frequent reviewer invitations, being up-to-date with research developments, opportunities to influence science, increased acumen in reviewing, free journal access or subscription, access to databases/research platforms and digital libraries, acknowledgment in journal websites, publicized reviews, letter of thanks, certificates of excellence, and editorial board appointment.’
Crediting	Barroga (2020)	‘Crediting incentives may be given by formally recognising the reviewing work and linking peer review activity to ORCID records using DOIs.’
Financial	Barroga (2020)	‘Financial incentives can be received through the Rubriq system by providing pre-publication reviews or from compensation derived from the article processing charge. Although cash incentives can hasten reviews, many journals cannot realistically afford it. Cash incentives may also affect the quality of review, transform the review process into business, or damage the moral sentiments of researchers. Other forms of financial incentives include waiver of publication charges and free access to paid articles.’
Reviewer credit	Tennant (2018)	‘How to provide and receive appropriate credit for peer review is an ongoing debate … There is … currently a great potential scope of providing more detailed information about peer review quality, in a manner that is further tied to researcher reputation and certification. The main barrier that remains here is the fact that peer review is still largely a closed and secretive process, which inhibits the distribution of any form of credit.’
Rewarding peer review	Burley (2017)	‘... recognizing and rewarding peer reviewers has become a priority for scholarly societies, publishers, and service providers. For example, societies publish lists of the most prolific and helpful reviewers; publishers give public credit and provide additional rewards; and service providers enable the collection of data on reviews and reviewers to enhance reviewer visibility and rewards. Further still, Publons is a start-up dedicated to publicly recognizing reviewers for their contribution, enabling reviewers to track and showcase their activities.
2. Reviewer support		
Label	Author	Description from paper
Guidelines and training	Barroga (2020)	‘Training is achieved when reviewing author instructions from journals, receiving guidance from academic peers, or continuing education on digitization and open access ... training and orientation through the Publons Academy can be received to further develop skills in reviewing.’
Core competencies	Barroga (2020)	‘Core competencies among peer reviewers are based on the recommendations of associations concerned with the integrity of peer review. These associations include the Council of Science Editors (CSE), World Association of Medical Editors (WAME), International Committee of Medical Journal Editors (ICMJE), and Committee on Publication Ethics (COPE). The core competencies commonly recommended by these associations may be categorized as reviewer's responsibilities to the authors, editors, and readers.’
Training and mentoring	Bruce *et al.* (2016)	‘Training, which included training or mentoring programs for peer reviewers to provide instructional support for appropriately evaluating important components of manuscript submissions. These interventions directly target the ability of peer reviewers to appropriately evaluate the quality of the manuscripts.’
Checklists	Bruce *et al.* (2016)	‘Peer reviewers’ use of a checklist, such as reporting guideline checklists, to evaluate the quality of the manuscript.’

***Technology to support peer review***

AI support for peer review, research discovery tools and publishing platforms amongst other technologies feature amongst the innovations described so far in this review. However, Barroga (2020) (after Tennant *et al.*, 2017) goes beyond the use of software tools and discusses possible future models of peer review based on particular types of technology. This section reports on these suggested future models. This is followed by Table 4 which summarises current use of technology and future models.

**Table 4.** Technology to support peer review.

1. Current uses		
Type	Author	Examples / Description from paper
Platforms/Servers/OA journals	Barroga (2020)	BMJ Open, Sage Open, PLOS ONE.
PR services	Barroga (2020)	Peerage of Science
Applications and Tools	Barroga (2020) and Burley (2017)	CrossCheck Publons
AI-assisted peer review	Barroga (2020)	‘Used for recognizing images, recommending content, detecting fraud, evaluating teaching and assessment, or detecting plagiarism; requires human final judgement’
2. Potential models		
Model	Author	Examples / description from paper
Reddit model	Barroga (2020); Tennant *et al.* (2017)	‘Platform for comments and original or linked content’
Stack exchange model	ibid	‘Network of websites of question and answer sites’
Amazon model	ibid	‘Model for posting reviews of published materials’
GitHub model	ibid	‘Open-source distributed version control system with features transferable to peer-review system’
Hypothesis model	ibid	‘Web annotation tool for interactive education and collection of peer perspectives’
Wikipedia model	ibid	‘Collaborative authoring and review system’
Blockchain model	ibid	‘Technology for possibly creating tokenized peer review system’
Hybrid peer review platform	ibid	‘Consists of harmonization, certification, and incentivization’

Barroga (2020) reviews the potential models of peer review put forward by Tennant *et al.* (2017) and assesses them against six features of open access publishing: openness, anonymity, accountability, bias, time and incentive. All the proposed models are open, in that review reports are public, but the identity of authors and reviewers remains unknown. On the factors of bias (whether editorial decisions are made public) and anonymity (whether the identity of editors and reviewers are revealed to authors) no assessment is made due to the models being hypothetical. The Reddit, Stack Exchange, and Hypothesis models are rated as offering greater author - reviewer accountability due to more transparent interactions between these stakeholders. Greater efficiency may be found in the GitHub and Wikipedia models with review time shortened or delays minimised. Reviewer incentives are found embedded within the Stack Exchange, block chain and hybrid peer review models.

## Discussion and summary

As review articles, the studies in our review draw conclusions based on several items of primary evidence. By bringing together these conclusions in a simple synthesis, it is possible to reveal some key messages, given that any similar conclusions drawn in the various review articles have the combined weight of all the primary evidence reviewed. The conclusions of the review articles have been integrated below, to highlight observations on current practice, perspectives on the implications of new ways of working and calls for action. The strongest conclusions, based only on frequency, are the need for more research to determine the effectiveness of new models of peer review, and the need for a full reflection on the peer review system, including all stakeholders.

### Observations on current practice

There are a number of observations summing up the current state of affairs in peer review: the increase in innovation has been rapid ( Barroga, 2020), there has been an overall improvement in reviewer recognition and efficiency of peer review processes, and an increase in initiatives trialing transparent peer review ( Burley, 2017). Technology is not being used to its full potential in the peer review system ( Tennant, 2018). Moving to reviewer focussed innovations, two of the reviews covered highlight the use of reviewer training, Burley (2017) commenting that scholarly publishers and learned societies are increasing their focus on training (and rewarding) reviewers, and Barroga (2020) suggesting that reviewer training and core competencies are important to consider as part of a broader reflection on the peer review system as a whole.

### Perspectives on the implications of newer practices

A number of authors discuss their interpretations on the implications of newer practices: that quality may be compromised if reviewers are paid ( Barroga, 2020); also, that innovations in peer review such as post-publication reviews and statistical reviews may reinforce a particular perspective in how scientific knowledge is perceived ( Horbach & Halffman, 2018). Rather than research outputs being seen as a snapshot of discovery, capturing one moment in time, which will be built on with new research, these innovations can lead to publications being edited with the aim of arriving at a set of inviolable facts. The authors suggest this is a new perspective, favoured by those with a realist or positivist view of knowledge perceiving the research literature as a ‘database of facts’ rather than a ‘library’ ( Horbach & Halffman, 2018, p. 13). Two reviews also cite specific barriers to change within the peer review system: that new models will never become mainstream whilst there is so much prestige to be gained from publishing in journals ( Tennant, 2018). In addition, new ways of working are often not in line with the behaviours required for job security and career progression, which results in very little motivation for researchers to change ( Tennant *et al.*, 2017; Tennant, 2018).

### Calls for action

Numerous calls for action are found in the conclusions drawn by the authors of the review articles. Three reviews ( Bruce *et al.*, 2016; Horbach & Halffman, 2018; Tennant *et al.*, 2017) conclude that there is a lack of empirical evidence to assess the effectiveness of innovations in peer review. On a more nuanced but similar point, Barroga (2020) and Horbach & Halffman (2018) point to the role of peer review to identify malpractice or errors in research, with Barroga calling for research to measure how far new innovations can deliver this, and Horbach and Halffman highlighting the tension between the practice of peer review and its ability to fulfil this role. Another conclusion that is shared is the need for a wider reflection on the peer review process as a research community, with both Barroga (2020) and Tennant (2018) underscoring the need to consider what different stakeholders bring to the peer review process and which role they inhabit. Tennant (2018) goes on to suggest that alongside appropriate use of technology and research incentives this reflection is necessary for the success of a new system of peer review. Finally, Tennant *et al.* (2017) call for the decoupling of peer review from commercial interests in order to return to a community-led process. Table 5 provides a summary of innovations described in this review.

**Table 5.** Summary of innovations. OA=open access.

	Review article					
Innovations	Barroga (2020)	Bruce *et al.* (2016)	Burley (2017)	Horbach & Halffman (2018)	Tennant (2018)	Tennant *et al.* (2017)
Open peer review	✓	✓		✓	✓	✓
Transparent peer review			✓			
Blinded / masked peer review		✓				
Pre-peer review commenting	✓					✓
Pre-publication peer review	✓					✓
Post-publication peer review	✓					✓
Post-publication commenting	✓					✓
Post publication peer review				✓		
Post publication peer review			✓			
Registered reports				✓		
Collaborative review	✓					✓
Interactive peer review	✓					✓
Discussion during review				✓		
Cascading peer review	✓		✓	✓		✓
Peer review as a separate service			✓			
Recommendation services	✓					✓
Portable review	✓					✓
Independent peer review	✓					✓
Decoupled post-publication review	✓					✓
Review by third parties				✓		
Specialisation		✓		✓		
Results free			✓			
Soundness only				✓		
Non-financial	✓					
Crediting	✓					
Financial	✓					
Reviewer credit					✓	
Rewarding peer review			✓			
Guidelines and training	✓					
Core competencies	✓					
Training and mentoring		✓				
Checklists		✓				
Platforms/servers/OA journals	✓					
PR services	✓					
Applications and tools	✓		✓			
AI-assisted peer review	✓					
Reddit model	✓					✓
Stack exchange model	✓					✓
Amazon model	✓					✓
GitHub model	✓					✓
Hypothesis model	✓					✓
Wikipedia model	✓					✓
Blockchain model	✓					✓
Hybrid peer review platform	✓					✓

## Conclusion

This review of innovations in peer review is based on papers identified in Web of Science and Scopus, limited from 2010 to 2021. A total of 247 papers were screened, with six recent review articles being included. These review articles comprise a mixture of narrative reviews, meta-analysis, state of the art and summary articles. They describe numerous approaches to peer review. In our meta-summary we collated these descriptions of peer review into four subcategories: open/masked; pre/post publication; collaboration and decoupling; focussed and specialised. We also collated mentions of reviewer focussed initiatives and presented these in the subcategories of reviewer support and reviewer incentives. We recorded and extracted references to the use of technology to aid peer review and summarised these practices noting current uses and potential models as reported in our included papers.

The fact that there are enough review articles to warrant a review of reviews, indicates the growing maturity of the field of peer review research. One review focussed on efficacy of peer review methods in a particular field ( Bruce *et al.*, 2016), and effectiveness evidence, testing and measuring how well particular innovations meet their objective continues to be a growing form of research in the field. However, given the size of the field and the inherent complexity of analysing the peer review system, which spans numerous disciplines and includes varied professions in its conduct, descriptive research in any form will always be essential to record the development in innovations. This meta-summary is a contribution in this vein. We hope that our overview of peer review innovations will support future work in this area.

## Data availability

No data are associated with this article.
