# Supplementary material for: An overview of innovations in the external peer review of journal manuscripts
Source: Wellcome Open Res. 2023 Jan 27;7:82. Originally published 2022 Mar 9. [Version 2] doi: 10.12688/wellcomeopenres.17715.2 (PMC9984734; doi:10.12688/wellcomeopenres.17715.2)
Supplement: Peer review history

# Peer review: Reviewer response for version 2

**DOI:** 10.21956/wellcomeopenres.20892.r54540

**Published:** 2023-03-03

**Authors:** Bahar Mehmani

**Affiliations:** 1 STM Journals, Elsevier, Amsterdam, The Netherlands

I thank the authors for adjusting their manuscript to address my previous concerns and comments. I do not have any further comments.

Is the review written in accessible language?

Yes

Are all factual statements correct and adequately supported by citations?

Yes

Are the conclusions drawn appropriate in the context of the current research literature?

Partly

Is the topic of the review discussed comprehensively in the context of the current literature?

Partly

Reviewer Expertise:

Peer review studies, research in peer review, peer review innovation

I confirm that I have read this submission and believe that I have an appropriate level of expertise to confirm that it is of an acceptable scientific standard.

# Peer review: Reviewer response for version 2

**DOI:** 10.21956/wellcomeopenres.20892.r54541

**Published:** 2023-01-30

**Authors:** Mario Malički

**Affiliations:** 1 Stanford University, Stanford, USA

Thank you for the updated version, but for the effort I invested in the initial review, I expected a point by point response to the raised comments. The naming/classification I see has remained as before without effort to align this with STM or other initiatives, the inclusion of a narrative summary, a paper without references remains questionable as any other research paper or opinion paper might have mentioned more "innovations" than that one, and future potential models remain included with too weak a rationale behind it as proposals listed in many papers on peer review could have fallen under the definition of potential future models.

Is the review written in accessible language?

Yes

Are all factual statements correct and adequately supported by citations?

No

Are the conclusions drawn appropriate in the context of the current research literature?

No

Is the topic of the review discussed comprehensively in the context of the current literature?

No

Reviewer Expertise:

Peer review

I confirm that I have read this submission and believe that I have an appropriate level of expertise to state that I do not consider it to be of an acceptable scientific standard, for reasons outlined above.

# Peer review: Reviewer response for version 2

**DOI:** 10.21956/wellcomeopenres.20892.r54539

**Published:** 2023-01-30

**Authors:** Erik Cobo

**Affiliations:** 1 Professor, Department of Statistics and Operations Research, Universitat Politecnica de Catalunya, Barcelona, Barcelona, Spain

The authors have responded to my comments and those of the other reviewers. The article has improved and, in my opinion, should be indexed.

Is the review written in accessible language?

Yes

Are all factual statements correct and adequately supported by citations?

Yes

Are the conclusions drawn appropriate in the context of the current research literature?

Yes

Is the topic of the review discussed comprehensively in the context of the current literature?

Yes

Reviewer Expertise:

Statistician-methodologist expert in pivotal clinical trials; and with experience in editorial boards

I confirm that I have read this submission and believe that I have an appropriate level of expertise to confirm that it is of an acceptable scientific standard.

# Peer review: Reviewer response for version 1

**DOI:** 10.21956/wellcomeopenres.19603.r49098

**Published:** 2022-06-08

**Authors:** Bahar Mehmani

**Affiliations:** 1 STM Journals, Elsevier, Amsterdam, The Netherlands

Thank you for the opportunity of reviewing this insightful review.

 Woods *et al. *have analyzed the body of published 'peer-reviewed' papers on innovation in peer review in scholarly publishing and report their findings in this paper. 

 It is important to note that by default innovation entails failure. No results and negative results of innovative ideas organized by journal publishers and societies as well as funders might have a hard time ending in the body of published literature if ever tried for and as such, it is even more relevant to consider non-peer-reviewed articles for such studies.

 Nevertheless, while the title and abstract mention innovations in scholarly publishing authors only discuss innovation in the process of reviewing journal manuscripts, and even there, only the external peer-review process through the lens of 4 previous reviews whose authors had the same focus. In this way, authors miss the opportunity to list innovations within the wider editorial process during the peer review, innovative ideas in books and monographs peer review, grant proposal peer review, and research elements such as data, code, protocol, software, and hardware peer review. 

 Also by limiting the search strategy to peer-reviewed and published in indexed journals, authors miss a few innovative ideas published by journals and publishers that do not have an incentive to publish a peer-reviewed paper about their initiative. These stakeholders usually announce their new initiatives on their web pages, or as blog posts. Below you will find a few examples.

 Examples of some other innovations in peer review that have not been listed in this paper are: 

- crowd review: 
https://www.thieme.de/en/thieme-chemistry/select-crowd-review-136859.htm
  and
https://filestage.io/select-crowd-review/
- reviewer monetary rewards: 
https://www.elsevier.com/authors/policies-and-guidelines/submission-fees
- co-reviewing:
https://systematicreviewsjournal.biomedcentral.com/about/peer-review-mentoring
  and 
https://www.elsevier.com/connect/reviewers-update/two-heads-are-better-than-one-working-with-a-co-reviewer
- Volunteering to review:
https://www.asabe.org/VolunteerToReview
  and
https://www.elsevier.com/connect/reviewers-update/our-reviewer-volunteer-journey

to name a few of the reviewer-focused innovations

 There is also a missed opportunity of narrowing the focus only to innovations in the external peer-review process. There have been several innovative streams to improve the editorial side of the peer review process that help speed up the process and improve the quality of feedback. A good example is using AI for matching manuscripts with reviewer areas of expertise resulting in several reviewer recommender tools such as:

- https://www.springernature.com/gp/editors/resources-tools/reviewer-finder
- https://www.elsevier.com/connect/editors-update/a-helping-hand-with-finding-reviewers-introducing-the-elsevier-reviewer-recommender

To summarize: 

 I understand addressing the above critics means changing the methods and scope of this study which is not preferable. I suggest authors change their title and abstract to reflect the above-mentioned items and add a limitation section to their paper listing these limitations.

Is the review written in accessible language?

Yes

Are all factual statements correct and adequately supported by citations?

Yes

Are the conclusions drawn appropriate in the context of the current research literature?

Partly

Is the topic of the review discussed comprehensively in the context of the current literature?

Partly

Reviewer Expertise:

peer review studies, research in peer review, peer review innovation

I confirm that I have read this submission and believe that I have an appropriate level of expertise to confirm that it is of an acceptable scientific standard, however I have significant reservations, as outlined above.

# Response

**Published:** 2023-01-06

**Authors:** Helen B Woods

Thank you for taking time to review this article and to present additional experiments with peer review in scholarly publishing not included in this review. Whilst these specific experiments were not included in our review, some of the approaches are included in a generic way. For example, the idea of co-reviewing or mentoring a new reviewer is a training intervention and we include some examples of this kind of innovation in the ‘Reviewer support’ section. In a similar way ‘Select Crowd Review’ Is an example of collaborative review. Some of the other examples represent approaches that are not described in our review, and this has been stated in the limitations section. In addition, innovations in other areas of the publishing process, such as speeding the efficiency of matching reviewers with manuscripts have not been included as you point out. We have made a statement to this effect in the limitations section to make this clearer to the reader and make a more transparent link between our data sources and the types of interventions found and included in our article.

# Peer review: Reviewer response for version 1

**DOI:** 10.21956/wellcomeopenres.19603.r50488

**Published:** 2022-05-26

**Authors:** Mario Malički

**Affiliations:** 1 Stanford University, Stanford, USA

Thank you for the opportunity to review this manuscript. I struggled with the paper a lot, and you will see below why. To me, this text read like an internal report, and not as a scholarly manuscript with elements and structure found in typical systematic, umbrella, or narrative reviews. It was also not clear what is your main contribution – a summary of terms you created and defined or a summary of terms others used. I expected the former.

-  Title – the meta-summary term should be avoided. If this is an umbrella review, use that, or specify why this is not one.

**Abstract**

- *quality assurance* – I recommend deleting this term or specifying in the methods/introduction what falls under this term and how it differs or overlaps with peer review. If it is important it should be included in the title – e.g. Overview (or umbrella review) of innovations in peer review and quality assurance in sc. publishing. If as you say later it's captured by your definition, avoid it here – as it implies it is not captured.  
- *This literature review* – why then use the term meta-summary in the title
- *The aim of this literature review* – please rephrase, and say what is the aim of this paper. Its relation and origin, and later use for the project can be described in the introduction/methods.
- Please rewrite the methods, and include information expected for lit/sys. review, who screened, how, in which software, language limits, and so on, as well as how, was summary conducted, was there a meta-analysis, and so on.
- Say our initial strategy found that all individual interventions we identified were captured in 6 reviews (and even some we did not), and so we adapted this project to be an umbrella. We grouped the intervention into….
- *From a simple synthesis of the review authors’ conclusions* – please mention first in the methods how you synthesized results and defined high level categories. I also hoped you would tell us which interventions were most commonly attempted.

**Introduction**

- I recommend a complete rewrite and expansion of the introduction, see comments on the abstract. What is the aim of this paper? The aims of the overall project, and how it came to be are secondary.  

**Definitions and framing.**

- I have difficulties with your definition of peer and would require it to be changed. A peer (reviewer) is an individual, not a software, or an AI. I understand that for the purpose of the article, and that in other published articles, the peer review term is used to describe all checks/suggestions (including those done by software) but that does not mean that the word peer needs to be redefined. Peer review is a compound noun, and its meaning can incorporate what you need, without redefining the word peer. Furthermore, later in the article, you use the term reviewer focused initiatives – which implies reviewer, is not used interchangeably with peer reviewer, nor does it include your peer definition.
- *Similarly, peer review also includes informal responses, questions, and comments posted on social media* – this goes against the new taxonomy of peer review – which specifically differentiates comments from peer review. So please state, in our study peer review also incorporates …
- *These types of informal responses were found in a study examining disciplinary knowledge production ( Woods, 2018), where examples of researchers’ peer review practice were identified*. – Please rephrase this I did not understand the meaning of this sentence. Did you want to say- those informal, or social media comments are similar to comments seen in peer review? See our study on preprint comments ^1^
- *Particularly in applied fields, this is coming to fruition*, - please provide a reference
- *This literature review describes innovations* – would recommend deleting this sentence, it is redundant
- *The next section will describe the methodology used in this literature review and how we will present the results. This is followed by the results themselves in which we classify different types of innovations. We conclude with some discursive reflections on the current situation*. – Would recommend deleting this, the subtitles below are sufficient

**Methods**

- *This review was undertaken* - please use the same term everywhere, and move this sentence to the background.
-  I find that the whole first section - Overall approach should be deleted, and search of literature stated first. Statements like - , it was not necessary to identify – if the paragraph is not deleted I recommend it is changed to:  We felt it was not – this is an issue or a limitation that a reviewer can oppose, and I would. – We did not limit the search – please first define, what you did search for and when, and provide a full search strategy. And then explain inclusion, and exclusion criteria, as well as bias assessment, or check to see how those papers matched your definition of peer review and innovations. If none were excluded, then state here that you included all papers that described any type of intervention/innovation.
-  Details of Reimagine Review are not necessary and should be deleted. Just cite it and say how many of their intervention made it to your final inventory.
-  Data collection – I would recommend using PRISMA 2020 subtitles, i.e. information sources, and that you use all applicable subtitles despite this not being sys. review You did use the Flow chart.
- If as you say in the end review articles were only included, then perhaps umbrella review is the best term
-  Presentation of results – would recommend deleting this paragraph
-  I find the description of 6 reviews unnecessary and would move them to the appendix. It is also not clear from the methods, what is the difference between a summary article, state-of-the art review, and narrative review – are these terms the authors used or you, how would you classify them, and why?
- Open/masked peer review – You could consider using the STM taxonomy rather than open/masked - https://osf.io/68rnz/
- In pre and post publication commenting, this amounts to comments or feedback by any interested party, - this is not true, there are specialized preprint review platforms actively inviting specific individuals. What would you call the platform where your article was posted and is peer-reviewed by me – who was invited to review?
- Registered reports or study protocols in my view should not be a sub-category under pre-post pub commenting. They are calls for review of study designs/ideas, rather than completed studies. And they can happen either as a pre-or post, as open or masked, as collaborative, or as decoupling. As can grant peer review. This makes me also question the term approach to peer review – what is approached here? In Burley's paper, she calls these models of peer review. How do other reviews call these innovations? Types, modes, approaches, descriptors? These approaches are descriptions of elements of peer review, which can one day be meta-data for peer review. A registered report is a type of article, not peer review.
- Looking at the titles of these categories, I fear a lot has been missed by not using the STM approach for subtitles, i.e. I would rename your “approaches” to Review focused Innovations or Review process interventions, and would call the sub-categories: Reviewer identity (innovations), Review timing (innovations), Reviewer interaction (innovations), Content focus (innovations),
- Technology support – rather than Technology to support peer review. – but see my comment 15
- As table 1 includes 576 included studies, I also hoped to see a table with these 576 studies classified by which innovations they cover. Was information based only on summaries in the 6 studies, without checking if something was missed by the original authors?
- Reviewer focused initiatives +- should rather be Reviewer focused innovations. But I believe you need to mention that AI checks and software cheeks are also Editor/publisher focused and can also be given to authors to self-evaluate their manuscripts. Like language software, self-evaluation checklists, statcheck, and so on.  Incentives can also be used to make editors do a better job. You also stated: that Barroga (2020) ^2^ cites informal training that researchers do for themselves – this is then an intervention on authors. So perhaps this category should be revised, and if the first high level is focused on the review process, then these here are perhaps focusing on review skill/expertise.  
- I find the weakest part of the summary to be the technical support, and the names assigned to them. GitHub and many other models are already captured in the “approaches section” they are rather platforms where a manuscript or peer review reports could be stored. And so there is nothing in the GitHub model that was not covered before. Amazon model refers to the rating of reviews – and this belongs to reviewer-focused initiatives not here. It should have been made clear when technology is used to enable other approaches, versus when it provides a new type of innovation. I find it also very strange that AI approach was listed primarily under focused review. Why does it matter who performs it? AI review can be pre-or post, it can be open or masked (in theory, if the type of AI used is not disclosed), it can be focused or for all. Why isn’t AI also mentioned under reviewer support, they can save time for reviewers to check many things. Finally, why use Baraga’s models and summarize them here. Can they be called an innovation, if they were never attempted in peer review– e.g. GitHub model, and amazon model. They are theories/models. You stated “what makes an innovation is if the practice is new to those who are implementing it. – if it was not implemented at least once, it should not have been included. It is a theory/model. What is it in this model that is not an innovation captured elsewhere? Just the use of a different term?
- My last comment is not directly about this paper, but more on the 4 schools described in How to improve scientific peer review: Four schools of thought - https://osf.io/preprints/socarxiv/v8ghj/. The categorization of high-level innovations here, and the schools showcase quite a different approach to classifications, and maybe both need to be revised in light of one another and my comments here.   

I will refrain from commenting on the discussion as I believe my above comments are already extensive and ask for a lot of changes, that should then also be reflected by rewriting the discussion.  

 In hopes that my comments may help you improve your manuscript,

 Mario Malički

Is the review written in accessible language?

Yes

Are all factual statements correct and adequately supported by citations?

No

Are the conclusions drawn appropriate in the context of the current research literature?

No

Is the topic of the review discussed comprehensively in the context of the current literature?

No

Reviewer Expertise:

Peer review

I confirm that I have read this submission and believe that I have an appropriate level of expertise to state that I do not consider it to be of an acceptable scientific standard, for reasons outlined above.

## References

[1] : Analysis of single comments left for bioRxiv preprints till September 2019. *Biochem Med (Zagreb)* .2021;31(2) : 10.11613/BM.2021.020201 020201 10.11613/BM.2021.020201 33927548PMC8047782

[2] : Innovative Strategies for Peer Review. *J Korean Med Sci* .2020;35(20) : 10.3346/jkms.2020.35.e138 e138 10.3346/jkms.2020.35.e138 32449322PMC7246191

# Response

**Published:** 2023-01-06

**Authors:** Helen B Woods

Thank you for reviewing our article and for alerting us to areas for improvement. What was particularly useful was to highlight instances where our use of language was less precise than it could have been, for example in the use of ‘peer review’, ‘peer’ and ‘innovation’. We have addressed these issues in the text. In response to other reviewers' comments we have added a limitations section and updated the title and abstract to more accurately reflect the scope of the article, which we think will also address some of your concerns regarding a more accurate description of the scope of the article. We have also provided additional citations in the text which have strengthened the article, for example to define the different review types discussed. With reference to the ‘Four Schools’ article, this has now been updated and is published here: https://osf.io/preprints/socarxiv/v8ghj/

# Peer review: Reviewer response for version 1

**DOI:** 10.21956/wellcomeopenres.19603.r49097

**Published:** 2022-04-22

**Authors:** Erik Cobo

**Affiliations:** 1 Professor, Department of Statistics and Operations Research, Universitat Politecnica de Catalunya, Barcelona, Barcelona, Spain

I’m thankful to be able to review the Wellcome OR manuscript titled "Innovations in peer review in scholarly publishing: a meta-summary”.

 I just have two major suggestions for authors. And one minor comment -that the authors might consider or not.

**Major suggestions**

- Please, on Methodology, provide operational definitions for the terms “meta-summary”, “narrative review”, “summary article” and “state-of-the-art review”.
- Please, revise the numbers on the flowchart. Note your actual numbers did not add (223 excluded +291 screened should equal 247 after duplicates are removed?).
- Please, consider further explanations on the figure foot or the text.

**Minor comment**

- Your text and your conclusion seem to avoid statements about the methods to provide evidence of the peer review processes. In particular, I wonder if there is any causal link between those peer review methods and the ultimate goal of Science, reproducibility.

Is the review written in accessible language?

Yes

Are all factual statements correct and adequately supported by citations?

Yes

Are the conclusions drawn appropriate in the context of the current research literature?

Yes

Is the topic of the review discussed comprehensively in the context of the current literature?

Yes

Reviewer Expertise:

Methodologist expert in pivotal clinical trials

I confirm that I have read this submission and believe that I have an appropriate level of expertise to confirm that it is of an acceptable scientific standard, however I have significant reservations, as outlined above.

# Response

**Published:** 2023-01-06

**Authors:** Helen B Woods

Thank you for taking the time to review our article, and for providing us with feedback to improve our work. This is much appreciated. We have addressed the major suggestions including a new simplified and accurate flow chart (hence no additional notes in a footer have been added to Figure 1). With reference to your comments about research methods and causal links, we have described the review papers that the article is based on, this includes the characteristics of each article, including research methods where these were employed. In our article, we didn’t set out to establish any links between reproducibility and particular types of peer review or particular methods to research peer review processes. However, the desire to improve the quality of research, including more detailed and accurate reporting, is clearly a motivating factor for many of the initiatives we describe.
